# Novel Pyrazine-Bridged D-A-D Type Charge Neutral Probe for Membrane Permeable Long-Term Live Cell Imaging

**DOI:** 10.3389/fchem.2021.782827

**Published:** 2021-12-01

**Authors:** Pei Liu, Suna Chen, Wenxuan Zhao, Qiutang Wang, Shuqi Wu, Liang Xu, Dan Bai

**Affiliations:** ^1^ Frontiers Science Center for Flexible Electronics (FSCFE), Institute of Flexible Electronics (IFE), MIIT Key Laboratory of Flexible Electronics (KLoFE), Xi'an Key Laboratory of Special Medicine and Health Engineering, Northwestern Polytechnical University, Northwestern Polytechnical University, Xi'an, China; ^2^ Research and Development Institute of Northwestern Polytechnical University in Shenzhen, Northwestern Polytechnical University, Xi'an, China; ^3^ Research Institute of Xi'an Jiaotong University (Zhejiang), Hangzhou, China; ^4^ Department of Chemistry and Chemical Engineering, School of Natural Sciences, Northwestern Polytechnical University, Xi'an, China; ^5^ School of Material Science and Engineering, Northwestern Polytechnical University, Xi'an, China; ^6^ School of Medicine, Xi'an Jiaotong University, Xi'an, China; ^7^ School of Life Sciences, Northwestern Polytechnical University, Xi'an, China; ^8^ School of Chemistry and Chemical Engineering, Key Laboratory for Green Processing of Chemical Engineering of Xinjiang Bingtuan, Shihezi University, Shihezi, China

**Keywords:** fluorescent probe, live cell imaging, pyrazine, D-A-D molecules, long-term imaging

## Abstract

A novel donor–acceptor–donor (D-A-D) type compound containing pyrazine as the acceptor and triphenylamine as the donor has been designed and synthesized. The photophysical properties and biocompatibility of this probe, namely (OMeTPA)2-Pyr for live cell imaging were systematically investigated, with observed large Stokes shifts, high photostability, and low cytotoxicity. Furthermore, we demonstrated that (OMeTPA)2-Pyr could permeate live cell membranes for labeling. The proposed mechanism of this probe was the binding and shafting through membrane integral transport proteins by electrostatic and hydrophobic interactions. These salient and novel findings can facilitate the strategic design of new pyrazine-fused charge-neutral molecular platforms as fluorescent probes, for long-term *in situ* dynamic monitoring in live cells.

## Introduction

Using fluorescent probes for long-term monitoring of live cells is the key to understanding and modulating molecular events happening within organelles, unraveling the physiological dynamics. Recently reported fluorogenic probes involve the D-A (donor–acceptor) type platforms such as substituted acetylnaphthalene derivatives (Tang and Jiang, 2017; Zhang et al., 2021a; Gao et al., 2021), BODIPYs (Tian et al., 2018; Bai et al., 2019; Liu et al., 2019; Zhang et al., 2021b; Li et al., 2021), AIEgens (Shi et al., 2019; Xu et al., 2021; Zheng et al., 2021), and organometallic compounds (Jin et al., 2021; Zhu et al., 2021; Ünlüer, 2021). However, tuning the bandgaps for predictable photophysical properties of D-A type molecules was limited by synthetic approaches. On the other hand, D-A-D type chromophores have currently attracted much research attention for showing great potentials as a fine-tuning platform for opto-electronic applications (Yang et al., 2020; Liu et al., 2021). In recent reports, D-A-D fluorescent probes have been used in imaging of lysosomal nitric oxide (Wang et al., 2018) and biothiol (Chen et al., 2018). With enriched variety of structure–activity relationships and a larger degree of conjugation that generates a longer wavelength emission, the D-A-D type molecular systems can reach desired performance indicators as probes through a rational design.

Under physiological microenvironments, aggregated caused quenching (ACQ) may happen that interferes the sensitivity and signal to noise ratio of fluorescent probes (Chen et al., 2020; Wang et al., 2021). Therefore, fluorophores with background-free signals are desired in bioimaging. It has been proven that in pure aqueous media, heteroaromatic-fused probes show none or weak fluorescence due to their formation of intramolecular H-aggregates, whereas in microenvironments with mixed organic media lipophilic and components, distinct fluorescence signals are obtained due to the unfolding and disruption of the probes’ single domain alignment (Kobayashi and Choyke, 2011; Lee et al., 2014; He et al., 2019). Considering choosing suitable aggregate moieties as functional groups in fluorescent probes, tetrenyl styrene (TPE), tetrenyl-1,4-dibutylene (TPBD), and distyrene (DSA) were reported susceptible to photothermal oxidation which leads to poor photostability, due to their strong electrophilic C-C double bonds that can react with nucleophilic reagents; the synthetic complexity of tetrenyl-1,4-dibutylene (TPBD) and hexabenzene (HPS) makes their scalability unfeasible for rapid accessible at a low cost (Chen et al., 2019; Xia et al., 2019; Cai and Liu, 2020). To tackle the above issues, in recent reports, a range of D-A-D type molecular systems have been reported. Lou et al. reported a D-A-D type curcuminoid-based fluorophore with a high signal to noise ratio (SNR) for biothiol recognition in living cells (Yang et al., 2020). Zhang et al. and Wang et al. reported fluorescent imaging *via* triphenylamine (TPA)-based probes (AS2CP-TPA, TTVP) with hydrophilic pyridinium salt moiety (Liu et al., 2020a), demonstrated a fast staining protocol. However, these probes were limited to stain the cell plasma membrane, thus, required additives such as lipid vesicle reagents (Morris et al., 2009; Suzuki et al., 2016; Han et al., 2017). Therefore, there is still an unmet demand for rational design and synthesis of novel probes with synthetic simplicity, tunable photophysical properties, greater stability, and biocompatibility (Liu et al., 2020b; Wang et al., 2020).

## Results and Discussion

### Preparation of (OMeTPA)2-Pyr Probe

**SCHEME 1:**
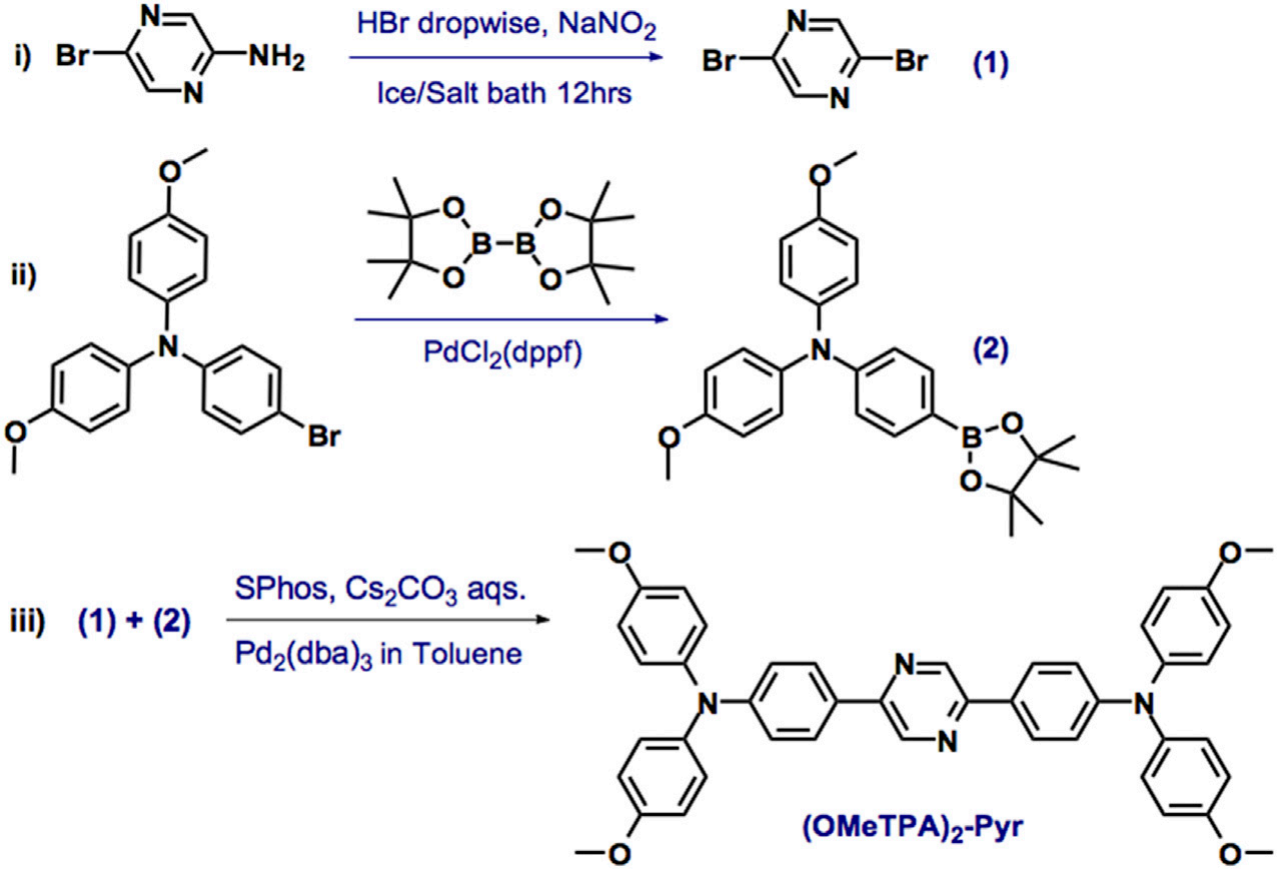
The synthesis of (OMeTPA)2-Pyr. Reagents and conditions: Pd2 (dba)3, SPhos [2-Dicyclohexylphosphino-2′,6′-dimethoxybiphenyl] and cesium carbonate aqueous solution in toluene, 80°C, 12 h.

In view of current challenges in probing molecular platforms, we have synthesized a D-A-D type probe with a rational design. The consideration for the molecular design is to retain photostability and imaging brightness by expanding the degree of π-conjugation and enhancing the intramolecular charge transfer effect through the D-A-D configuration. Pyrazine was chosen as the core acceptor moiety, which reacted with 4-methoxytriphenylamine as the donor group. The lone-pair electrons of the terminal O and N atoms in the TPA groups were able to efficiently delocalize the π-bonds. As the LUMOs are relatively concentrated on the pyrazine core, the intramolecular charge transfer (ICT) effect would be promoted upon excitation during the imaging process to ensure the photostability (Yang et al., 2020; Liu et al., 2021). Synthesis was carried out in a one-pot reaction by the Suzuki coupling reaction Pd2 (dba)3, SPhos, and cesium carbonate aqueous solution in toluene, 80 °C, 12 hrs (Scheme 1) in moderate yield as yellow powder. The correct chemical structure of (OMeTPA)2-Pyr was verified by 1H and 13CNMR spectra alongside mass spectrometry (Supplementary Figure S1–3) with satisfactory analysis.

### Density Functional Theory (DFT) Calculations

**FIGURE 1:**
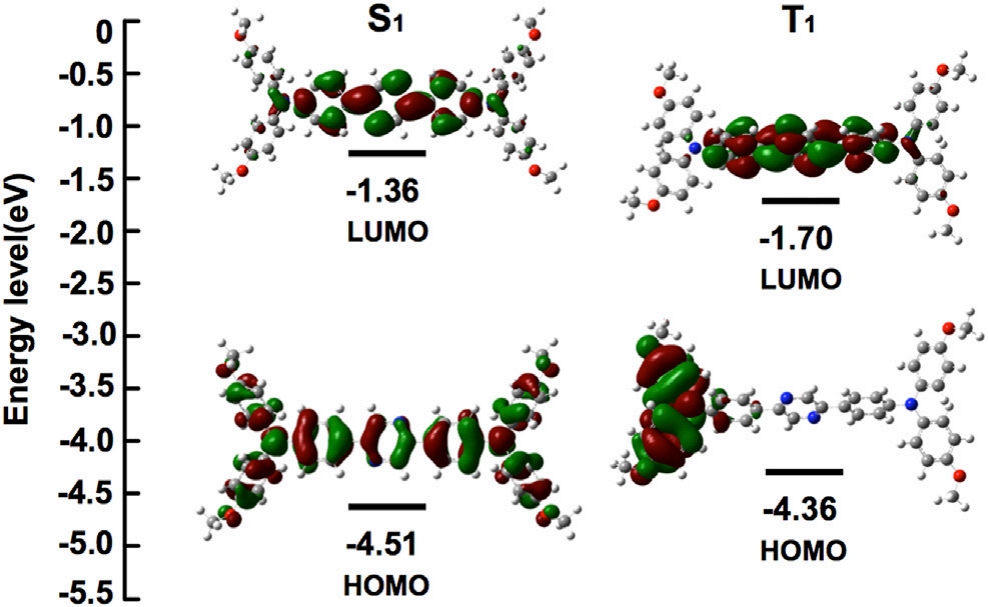
Energies and electronic orbital localizations of (OMeTPA)2-Pyr in its representative Frontier molecular orbital distributions.

Introduction of the bulky 4-methoxy-TPA substituents as donor on the pyrazine core as the acceptor orchestrated the electronic distribution and photophysics property of the fused D-A-D type compound (Figure 1). It was found that the horizontal dispersion of LUMO levels was mostly distributed on the central pyrazine core with the central π bridge; however, the electronic cloud of the HOMO levels in the compound could be delocalized further over the entire molecule. This electronic distribution was considered facilitating the inherent intramolecular charge transfer (ICT) characteristics of (OMeTPA)2-Pyr, which were further confirmed *via* spectroscopy.

### Photophysics Analysis

**FIGURE 2:**
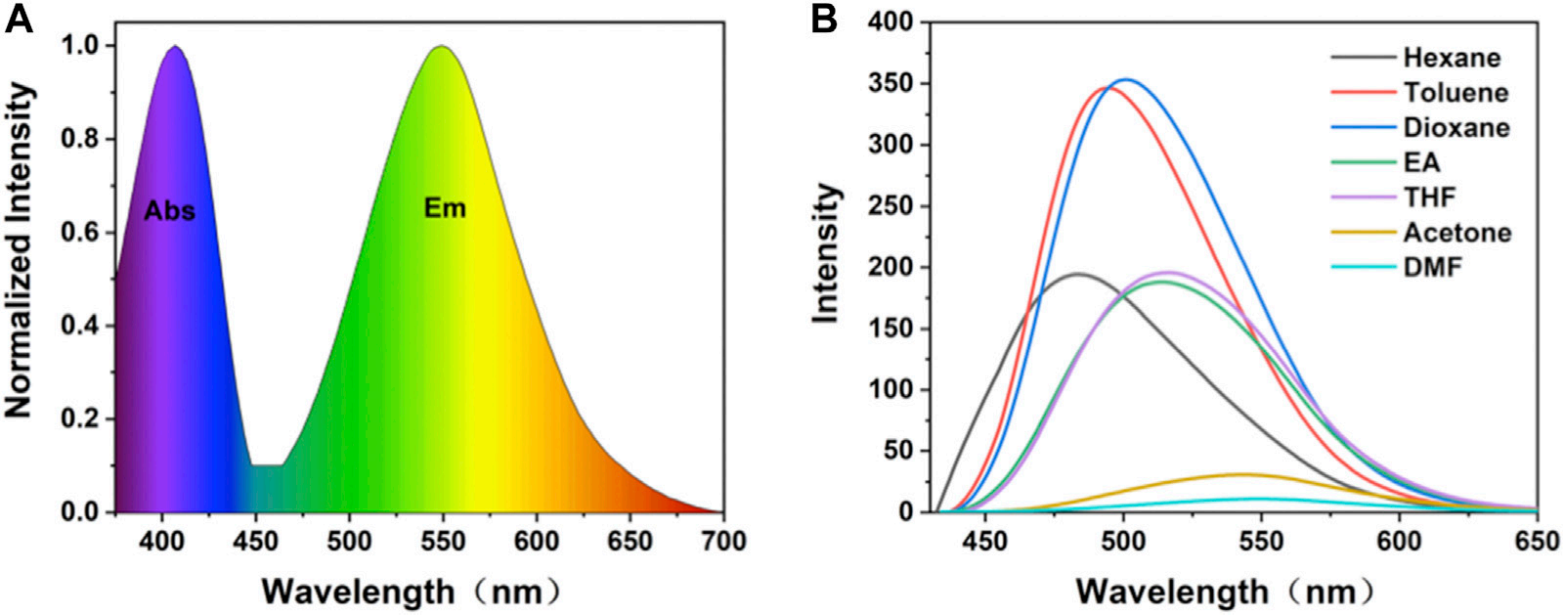
**(A)** Normalized absorption and fluorescence emission spectrum of (OMeTPA)2Pyr in DMF; **(B)** Fluorescence spectra of (OMeTPA)2Pyr in variant solvents (λex = 427 nm).

From spectroscopy, (OMeTPA)2-Pyr was found with a quantum yield ΦFL = 0.36 and lifetime τ= 1.42 ns in DMF. In accord with the DFT calculation, the spectra have shown the existence of an intramolecular charge transfer (ICT) excited state in the D-A-D system. Two absorption bands of 289 nm (n→σ*) and 416 nm (n→π*) were observed in the UV-Vis spectrum, which were relatively insensitive to polarity changes in solvents (Figure 2A, Supplementary Figure S4). The maximum excitation wavelength of (OMeTPA)2-Pyr was found at 427 nm (Supplementary Figure S5), and the fluorescence emission spectra were strongly affected by the polarity of surrounding solvents (Figure 2B). The emission peak of (OMeTPA)2-Pyr red shifted from 489 nm (in hexane, apolar aprotic) to 551 nm (in DMF, polar aprotic), respectively (Table S1). The sensitivity of (OMeTPA)2-Pyr in the solvent environment was estimated by measuring the difference in units of wavenumbers (Δν) between the maximum absorption and emission, deduced from the Lippert-Mataga equation (Chen et al., 2019) (Zhang et al., 2021a),

$$\begin{array}{l} {\bar{V}}_{Abs}-{\bar{V}}_{FL}=\Delta \bar{V}=\frac{2}{hc}\left(\frac{\varepsilon -1}{2\varepsilon +1}-\frac{n^{2}-1}{2n^{2}+1}\right)\frac{{\left(\mu_{\varepsilon }-\mu_{G}\right)}^{2}}{a^{3}}\\ =\frac{2\Delta f}{hc}\frac{{\left(\mu_{\varepsilon }-\mu_{G}\right)}^{2}}{a^{3}}, \end{array}$$ [1]

in which, νAbs is the wavenumber of the absorption, νF is the wavenumber of the fluorescent emission, h is Planck’s constant, c is the velocity of light, and a is the Onsager cavity radius around the fluorophore, which was calculated from the DFT optimized lowest energy structure. In this case, the Onsager radius a (0.68 Å) was taken as half the average distance between the 4-methoxyphenylaniline (-OCH3) in the donor moiety and the imine nitrogen carbon of the pyrazine acceptor, which correlates with the longest possible axis across the molecule where charge separation could take place; Δμ = (μE-μG) represents the difference between excited and ground states dipole moments. Δf is defined by the dielectric constant ε and the refractive index n, which represents the orientation polarizability. In accord with the Lippert–Mataga formula, the Stokes displacement is proportional to the orientation polarizability with different solvents, relevant to Δμ (the difference between the dipole moment between the excited state and the ground state). The D-A-D type structure with the planar TPA group and twisted molecular conformation made the (OMeTPA)2-Pyr probe possess both π-conjugation and intermolecular π-π interactions. Upon excitation, π electrons are easily polarized. The dipole moments in the excited state are known to be greater than theirs in the ground state. Hence, the excited energy of this molecule would be stabilized by the polar solvent to a greater extent, which correlates with the solvent dependency of Stokes’ shift changes. The Stokes’ shift measured in the variant solvent can be seen changing linearly in the Lippert-Mataga plot (Supplementary Figure S7, S8) in response to the solvent polarity. The Stokes shift of (OMeTPA)2-Pyr changed from 64 nm (3080cm-1) in hexane to 124 nm (5314cm-1) in DMF, respectively, correlating with Δμ (8.0 D), proportional to the orientation polarizability.

From the spectroscopy-measured excited-state dipole moment data alongside DFT calculation, the electron transfer process can be seen in this D-A-D molecular system. The obtained DFT values of redox potentials indicate that the subunits interact very weakly in the ground states. Introduction of electron-donating methoxy groups enhanced the electron push-pull effect and the conjugate level of the D-A-D molecule. Meanwhile, due to the rotation of the 2-phenylpyrazine single bonds between the donor and acceptor, activation of the ICT process was observed accompanying by conformational changes in molecular geometry and therefore, induced the formation of twisted intramolecular charge transfer (TICT) states apart from the excited state charge transfer (Supplementary Figure S9).

Considering the use of (OMeTPA)2-Pyr for live cell probing under physiological environments, the fluorescent property was then tested in the gradient mixture of DMF with water (protic solvent). Intensity of maximum fluorescence increased with water fraction range 30–70%. Dynamic light scattering measurement has found a smaller average diameter (19 nm) and a fewer polydispersity index (0.305) of the probe in aqueous solution than in DMF solvents (Supplementary Table S2, Figure S10). Attribute to the free rotation of methoxy groups with minimum electron affinity in the D-A-D axis plane orientations, the non-aggregate homogenous distribution property in the aqueous solution maketh the probe compatible for live cell imaging.

### Molecular Docking Simulations

**FIGURE 3:**
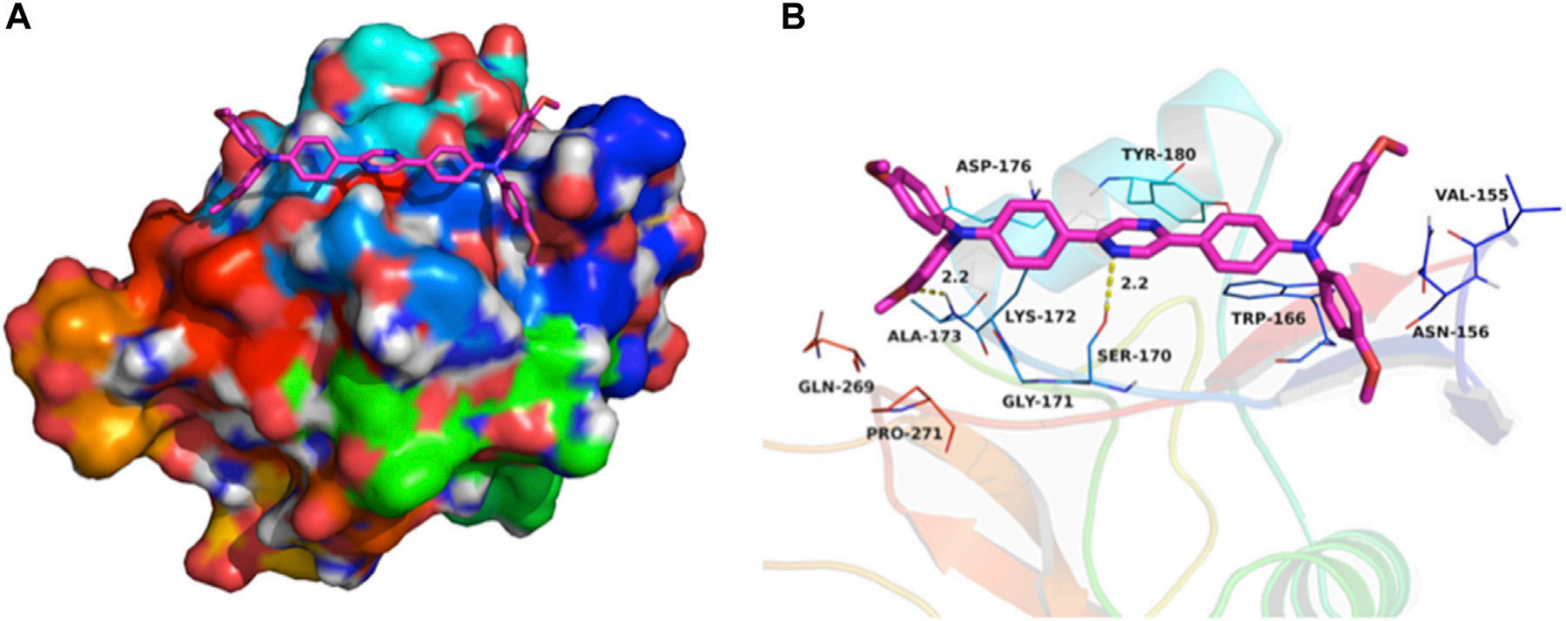
**(A)** Total view of; **(B)** Detailed view of (OMeTPA)2Pyr docked into the binding site of the ASGPR transmembrane glycoprotein. Representative binding residues were shown in blue/green lines; the probe molecule was represented with rose red sticks with N/O atoms highlighted; The hydrogen bonds were shown in yellow dotted lines.

Using the aromatic heterocycle with two nitrogen atoms, the pyrazine core could affect the protonation and hydrogen-bond formation with the membrane integral transport proteins and facilitate the live cell membrane permeability. To validate this hypothesis, (OMeTPA)2-Pyr was docked into the binding site of the human asialoglycoprotein receptor ASGPR (PDB ID: 1DV8, typeII membrane integral transport protein). The maximum binding affinity between (OMeTPA)2-Pyr and the ASGPR was predicted to be -5.6 kcal/mol. The probe adopted a compact conformation to bind at the site of the ASGPR (Figure 3A). The probe located at the hydrophobic pocket, surrounded by the residues Val-155, Trp-166, Ala-173, and Pro-271, forming a stable hydrophobic binding (Figure 3B). Detailed analysis showed that the phenyl groups of the (OMeTPA)2-Pyr formed cation-π and anion-π interactions with the residues Lys-172 and Asp-176, respectively. Importantly, two key hydrogen bond interactions were observed between the (OMeTPA)2-Pyr and the residues Ser-170 and Ala-173 (bond lengths: 2.2 Å and 2.2 Å), which was the main interaction between the (OMeTPA)2-Pyr and the ASGPR (Figure 3B). All these interactions facilitated the probe to anchor tightly into the binding pocket of ASGPR, rather than attach at the shallow surface of the peptide side chains. Therefore, this charge neutral probe was enabled to interact with membrane transporters for attained live cell permeability, which was then confirmed by time-resolved live cell imaging results.

### Live Cell Confocal Laser Scanning Microscopy Imaging

**FIGURE 4:**
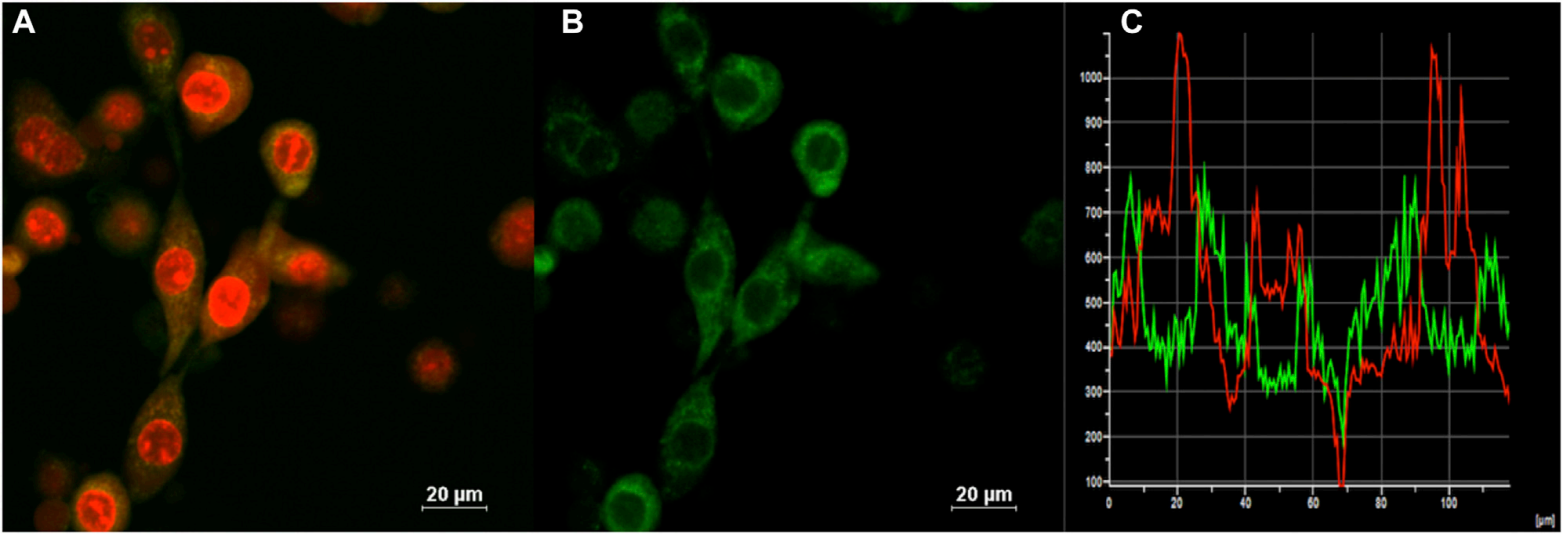
CLSM images of **(A)** Merged image of all channels for the cells co-stained with a commercial nuclear stain RedDot™(1.0 μM, 30 min) and probe; **(B)** (OMeTPA)2Pyr (2.0 μM with 0.1% DMSO in cell medium) treated live Hela cells after 30 min staining; **(C)** Calculation of co-localization coefficient. Scale bar: 20 μm.

**FIGURE 5:**
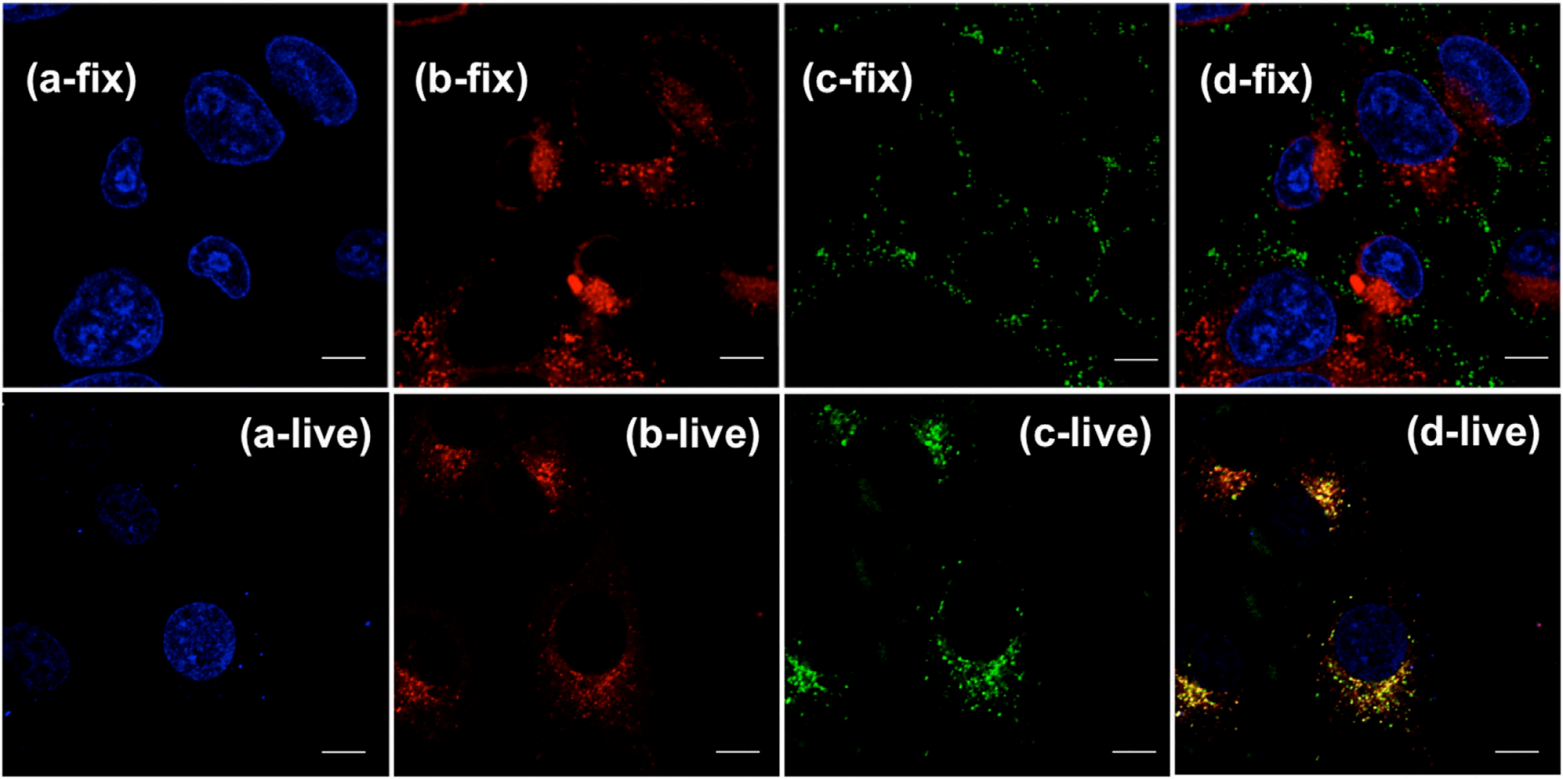
CLSM images of live Hela cell (a-/b-/c-/d-live) and DMF fixed cell (a-/b-/c-/d-fix) co-stained with commercial dyes and probe: **(A)** channels of nuclear stain Hoechst 33,342; **(B)** channels of mitochondria tracker MitoTracker^®^ Red; **(C)** channels of fixed and live cells stained with (OMeTPA)2-Pyr (1.5 μg/ml) in 24 h, respectively; **(D)** Merged image of all channels. Scale bar, 10 μm.

Confocal laser scanning microscopy (CLSM) imaging results (Figure 4) showed that the distribution of (OMeTPA)2-Pyr was mainly in live cell plasma, fewer at cell nucleus, none co-localizing with the commercial nuclear stain RedDot™ (Figure 4) nor Hoechst 33,342 (Figure 5); partially co-localizing with the commercial mitochondria tracker MitoTracker^®^ Red (Pearson correlation 35.7%). The linear fluorescence intensity was measured using ImageJ; the average diameter of the stained points was 1.27 mm with high uniformity (Supplementary Figure S11). (OMeTPA)2-Pyr was found to effectively label the cells with obviously homogenous distribution in the cytoplasm without leaching out from the stained cells over incubation time (24 h). This result suggested that (OMeTPA)2-Pyr is unlikely to cause false positive stain due to leakage. The membrane permeability and live cell-specific labeling of (OMeTPA)2-Pyr was then confirmed by imaging results in fixed cells (Figure 5). In 4% formaldehyde-fixed Hela cells, (OMeTPA)2-Pyr was stained solely on the cell membrane thitherto none uptake by the cytoplasm after 24 h incubation. In accord with the molecular docking simulations, hydrophobic pocket binding formation of (OMeTPA)2-Pyr with the asialoglycoprotein receptor (ASGPR)-a type II transmembrane glycoprotein played important role in its live cell cytoplasm localization.

**FIGURE 6:**
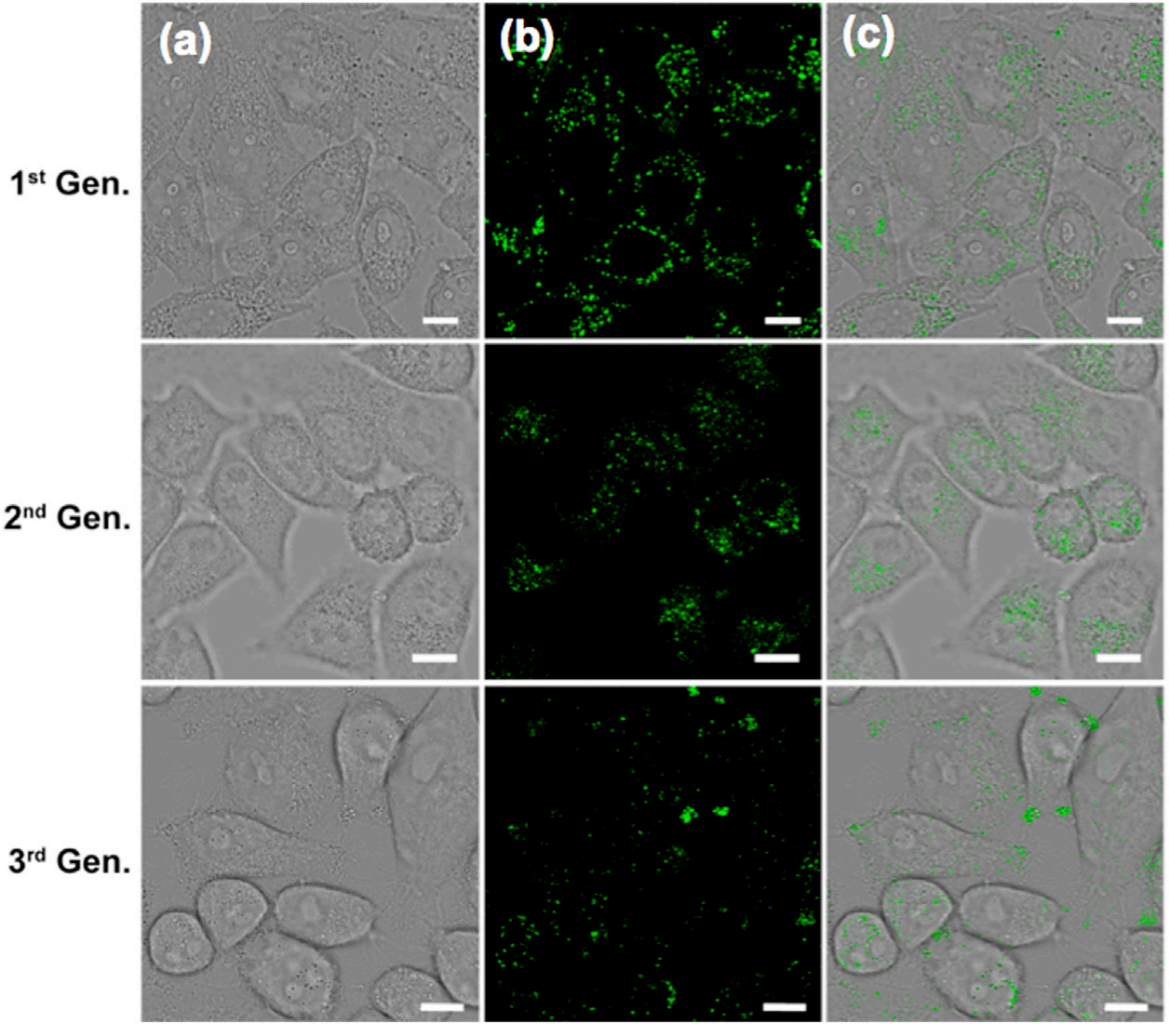
CLSM images of live Hela cells stained with (OMeTPA)2Pyr (1.5 μg/ml) over different cell generation. Scale bar, 10 μm.

The photophysical fidelity and biocompatibility of (OMeTPA)2-Pyr in live cell imaging were further examined. Photo-bleaching, which is a common challenge for many organic probes were not observed in this case. Live Hela cells precultured with (OMeTPA)2-Pyr were exposed to constant 405 nm laser illumination, the intracellular fluorescence intensity was recorded over time by confocal microscopy and calculated by ImageJ software. Results have showed that (OMeTPA)2-Pyr exhibited constant fluorescence emission and the relative intensity remained over 70% after 2 h illumination, suggesting the photostability of (OMeTPA)2-Pyr inside live cells (Supplementary Figure S13). The biocompatibility of (OMeTPA)2-Pyr was evaluated in HeLa and 4T1 cells by CCK-8 assay for 24 h incubation at a dosage up to 50 μg/ml (Supplementary Figure S14), which was over 30 fold higher than the concentration used in cell labeling. The data suggested that (OMeTPA)2-Pyr labeling did not affect cell viability. Moreover, the quality of fluorescence images retained over different generations of (OMeTPA)2-Pyr-labeled live cells. Results have shown that the probe could be carried over to the three generation of offspring cells with photophysical consistency (Figure 6).

## Conclusion

A pyrazine-bridged D-A-D type fluorescence probe, namely (OMeTPA)2-Pyr, was designed and synthesized. This charge neutral probe was found selectively binding to membrane transporter protein and thus, could be taken by live cells for cytoplasm imaging. The advantages of photostability and long-term labeling render great potential to develop a novel molecular platform for long-term live cell imaging.

## Methods

### Synthesis of (OMeTPA)2-Pyr

In a 50 ml Shrek tube, the mixture of 2,5-dibromopyrazine (242 mg, 1.01 mmol), 4-methoxy-N-(4-methoxyphenyl)-N-(4-(4,4,5,5-tetramethyl-1,3,2-dioxaborolan-2-yl)--phenyl)aniline (1.29 g, 3.04 mmol),Pd2 (dba)3 (127 mg, 0.14 mmol),SPhos (88 mg, 0.21 mmol), and Cs2CO3 (6.91g, 21.21 mmol) in toluene/H2O (2:1, 15 ml) was stirred at 85 °C for 24 h under the protection of N_2_. After cooling the solution to room temperature, the reaction mixture was poured into a 250 ml separatory funnel to separate the organic phase and using CH_2_Cl_2_ to extract the aqueous phase. Then, the combined organic phase was dried by MgSO_4_ and concentrated by rotavapor. At last, the residue was purified by a silica gel column (Hexane:DCM = 1:1) to afford (OMeTPA)2-Pyr as a yellow solid (402 mg, 58% yield). 1HNMR (400 MHz, CDCl3): *δ* = 3.81 (s, 12H), 6.86 (d, J = 8Hz, 8H), 7.01 (d, J = 8 Hz 4H), 7.11 (d, J = 8Hz, 8H), 7.85 (d, J = 8Hz, 4H), 8.91 (s, 2H) ppm; 13CNMR (100 MHz, CDCl3): *δ* = 55.46, 114.75, 119.69, 127.07, 127.17, 127.65, 140.19, 149.21, 149.98, 156.26 ppm. HRMS (EI) m/z: calcd for C44H38N4O4 = 686.29; found [M+1]+ = 687.2965.

### Density Functional Theory (DFT) Calculations

Highest occupied (HOMO) and lowest unoccupied (LUMO) Kohn–Sham orbitals were conducted by TDDFT/B3LYP/6-31G*(d,p) for C/H/O/N based on the optimized molecular geometries in the ground state and first excited state. The orbital energies shown in parentheses were established with DFT calculations using Gaussian 09 software package. The optimized geometry and space plots were visualized using Multiwfn and VMD software.

### Molecular Docking Simulation

Dock vina 1.1.2 was used to investigate the binding mode between (OMeTPA)2-Pyr and the membrane transporter human asialoglycoprotein receptor (ASGPR). The 2D structure of the (OMeTPA)2-Pyr was drawn and converted to a 3D structure by ChemBio3D Ultra 14.0 software. The 3D structure of ASGPR (PDB ID: 1DV8) was downloaded from the RCSB Protein Data Bank (www.rcsb.org). AutoDockTools 1.5.6 package (Cai and Liu, 2020) was employed to generate the docking input files. The ligand was prepared for docking by merging non-polar hydrogen atoms and defining rotatable bonds. The search grid of the ASGPR site was identified as center_x: 3.911, center_y: 17.995, and center_z: 32.857 with dimensions size_x: 39.75, size_y: 39.75, and size_z: 35.25. In order to increase the docking accuracy, the value of exhaustiveness was set to 20. For Vina docking, the default parameters were used if it was not mentioned. The best-scoring pose as judged by the Vina docking score was chosen and visually analyzed using PyMoL 1.7.6 software (www.pymol.org).

### Cell Imaging

Cells were seeded on 35 mm glass-bottom dishes (NEST) and incubated in DMEM culture medium at 37 °C in 5% CO_2_ overnight. The cells were cultured for 40 min in DMEM spiked with (OMeTPA)2-Pyr (1.5 μg/ml) and commercial trackers. The cells were washed with PBS (1 ml) and placed in fresh culture medium. Then, the cells were analyzed by confocal fluorescence microscopy (Leica SP8) using the following filters: λex = 405 nm and λem = 420–550 nm for (OMeTPA)2-Pyr signal, λex = 552 nm and λem = 587–657 nm for the Mitotracker Red signal, and λex = 638 nm and λem = 710–880 nm for the RedDotTM1 signal. The fluorescence signals of MitoRed, RedDotTM1, and (OMeTPA)2-Pyr inside cells were merged using Photoshop CS 5.0. The fluorescence values and distributions were analyzed by the software of ImageJ.

### Cytotoxicity Assay (CCK-8)

To determine the biocompatibility of (OMeTPA)2-Pyr against different cell types, 4T1 and HeLa cells were seeded in the 96-well plate with 5 × 103 cells per well and incubated overnight in DMEM culture medium. The cells were washed with PBS once and then incubated in fresh culture medium containing various amounts of (OMeTPA)2-Pyr (0, 10, 20, and 50 μg/ml) for 48 h respectively. After washing with PBS twice, the cells received CCK-8 analysis following the manufacturer’s protocols.

## Data Availability

The original contributions presented in the study are included in the article/Supplementary Material, further inquiries can be directed to the corresponding author.
