# Supplementary material for: Novel Pyrazine-Bridged D-A-D Type Charge Neutral Probe for Membrane Permeable Long-Term Live Cell Imaging
Source: Front Chem. 2021 Dec 1;9:782827. doi: 10.3389/fchem.2021.782827 (PMC8672416; doi:10.3389/fchem.2021.782827)
Supplement: Supplementary file 1 [file DataSheet1.doc]

**Supplementary Information**

**Novel pyrazine-bridged D-A-D type charge neutral probe for membrane permeable long-term live cell imaging**

Pei Liu^e^, Suna Chen^a^, Wenxuan Zhao^f^, Qiutang Wang^g^, Shuqi Wu^h^, Liang Xu^i^, Dan Bai*^abcd^

*a. Frontiers Science Center for Flexible Electronics (FSCFE), Institute of Flexible Electronics (IFE), MIIT Key Laboratory of Flexible Electronics (KLoFE), Northwestern Polytechnical University, Xi'an, CN 710072*

*b. Research and Development Institute of Northwestern Polytechnical University in Shenzhen, Northwestern Polytechnical University, Xi'an, CN 518057*

c. Xi'an Key Laboratory of Special Medicine and Health Engineering, Northwestern Polytechnical University, Xi'an, CN 710129

d. **Research Institute of Xi'an Jiaotong University (Zhejiang), Hangzhou,** CN 311215

*e. Department of Chemistry and Chemical Engineering, School of Natural Sciences, Northwestern Polytechnical University, Xi'an 710072, PR China*

*f. School of Material Science and Engineering, Northwestern Polytechnical University, Xi'an, CN 710072*

*g. School of Medicine, Xi’an Jiaotong University, CN 710061 h. School of Life Sciences, Northwestern Polytechnical University, CN 710072 i. School of Chemistry and Chemical Engineering, Key Laboratory for Green Processing of Chemical Engineering of Xinjiang Bingtuan, Shihezi University, CN 832003*

*Correspondence:* [*iamdbai@nwpu.edu.cn*](mailto:iamdbai@nwpu.edu.cn)

**Materials and characterization instrumentation**

The materials used for synthesis were all purchased from Innochem (Beijing, China). Cell viability assay (CCK-8) was purchased from Sigma-Aldrich Co. LLC (Shanghai, China). Lysotraker Red and RedDot^TM^ 1 were purchased from Invitrogen (China). ^1^H and ^13^CNMR spectra in CDCl_3_ were determined by a BrukerAVANCE 400 MHz Spectrometer, and mass spectrometry was determined by Waters QTof. UV-Vis absorption spectra were recorded on Thermo Evolution 220 spectrometre. Photoluminescence spectra were recorded on Hitachi F7500 spectrometre. Fluorescence lifetime spectrum and quantum yield were recorded on Holiba F-7000 FL spectrophotometer with Quanta-φSpectralon® integrating sphere with center-mounted cuvette sample holder, Ludox® TMA colloidal silica was used as reference. CCK-8 assay was performed on a platereader (Synergy HT, BioTek).


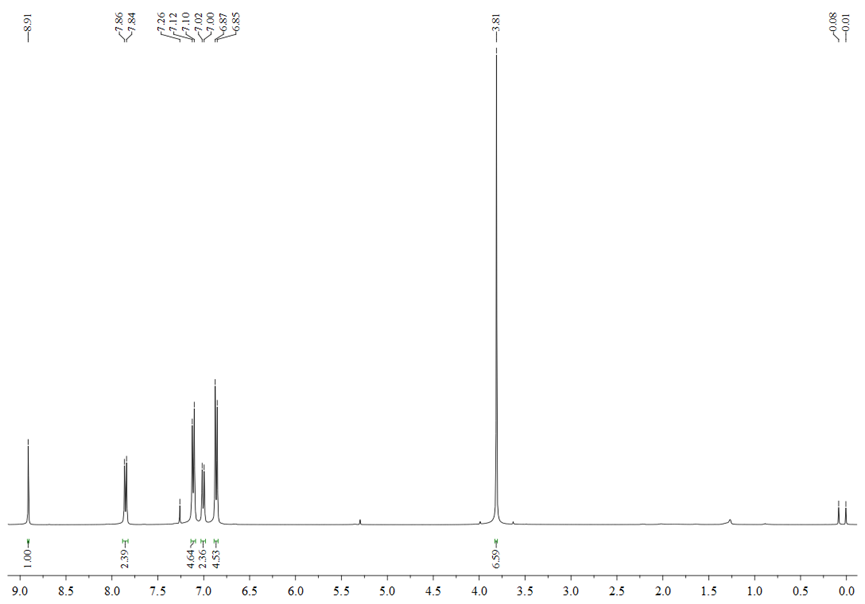


**Fig. S1** ^1^H NMR spectrum of **(OMeTPA)_2_**-**Pyr** in CDCl_3_.


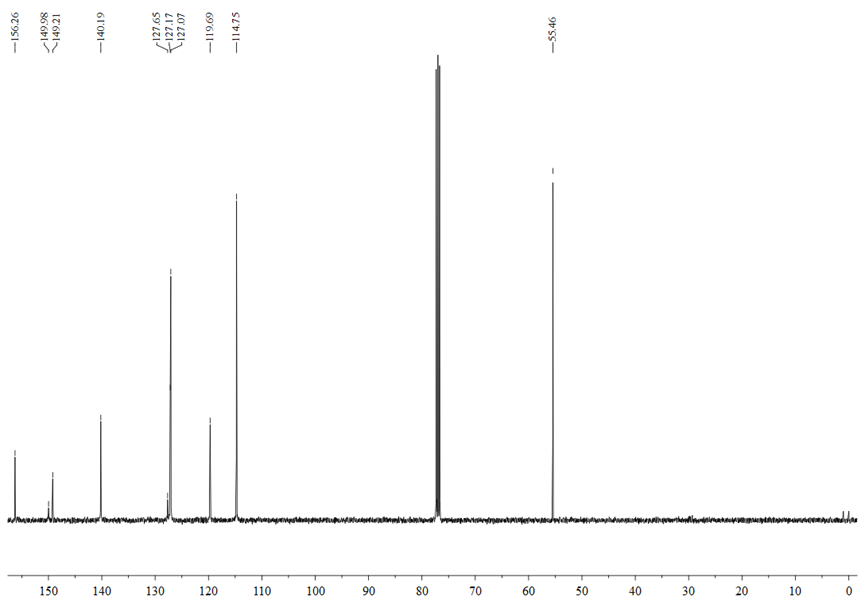


**Fig. S2** ^13^C NMR spectrum of **(OMeTPA)_2_**-**Pyr** in CDCl_3_.


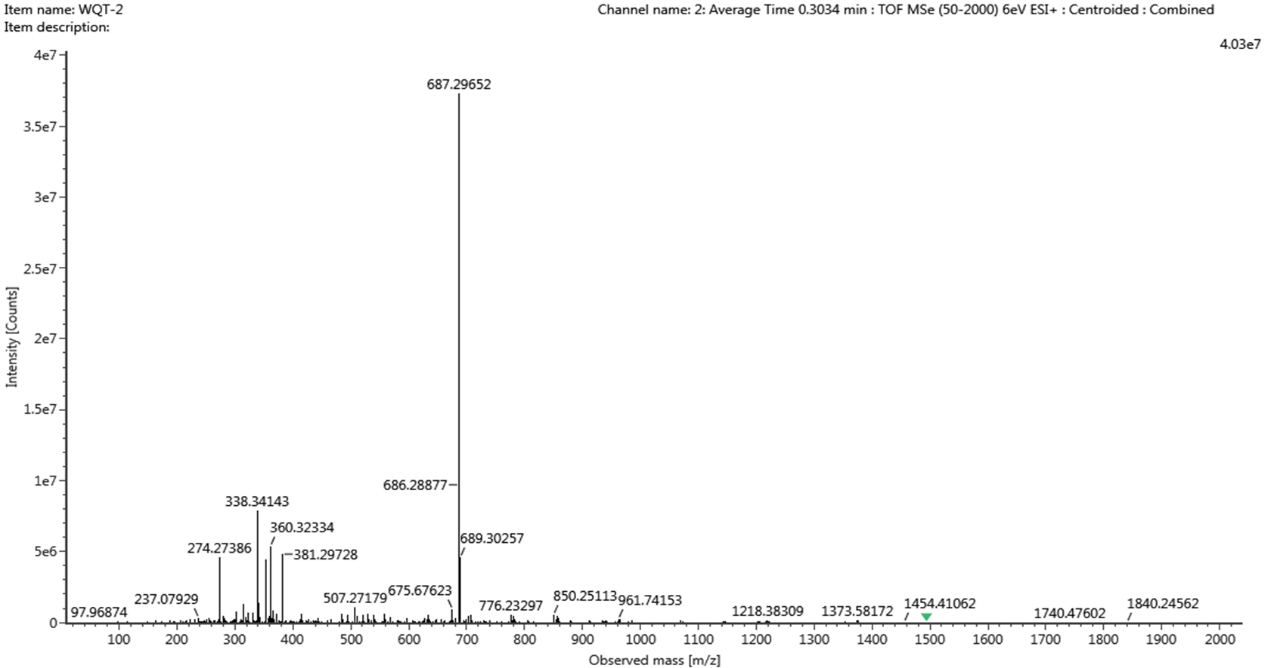


**Fig. S3** HRMS spectrum of **(OMeTPA)_2_**-**Pyr** in acetonitrile.


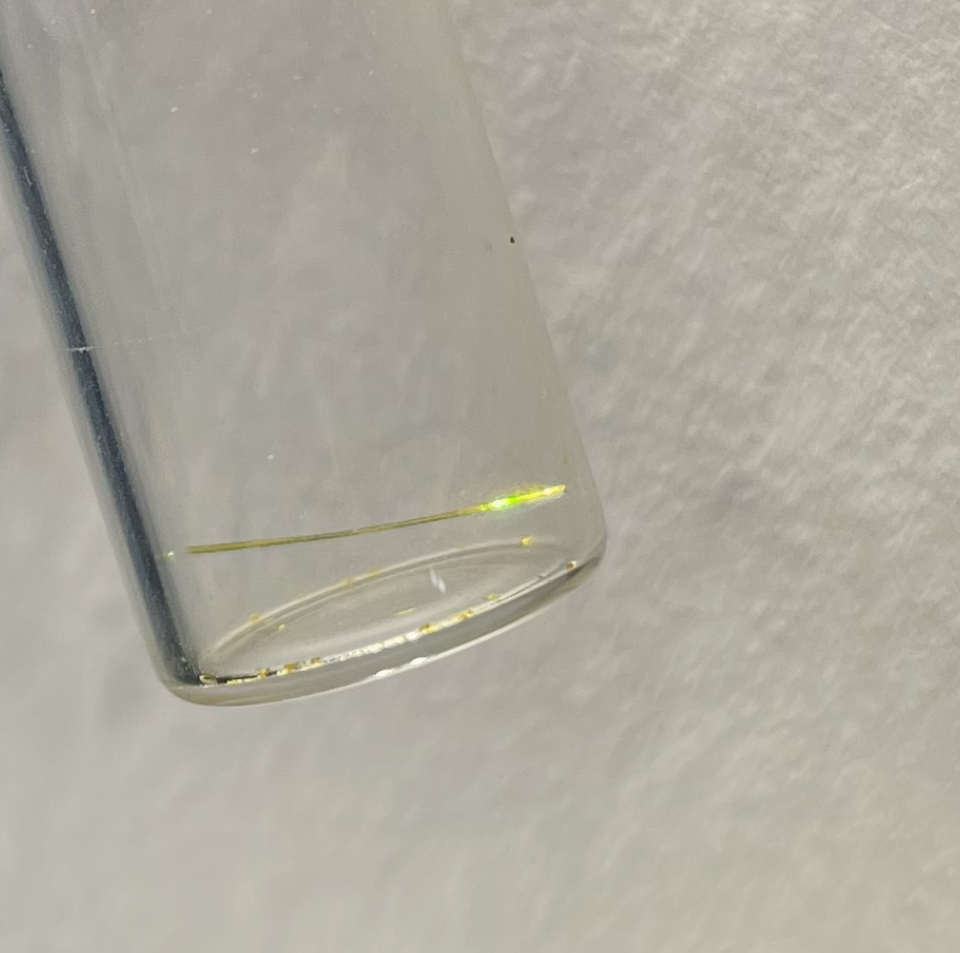


**Fig. S4** Single crystal of **(OMeTPA)_2_**-**Pyr** isolated by slow vapor diffusion (CHCl_3_:Hexane).


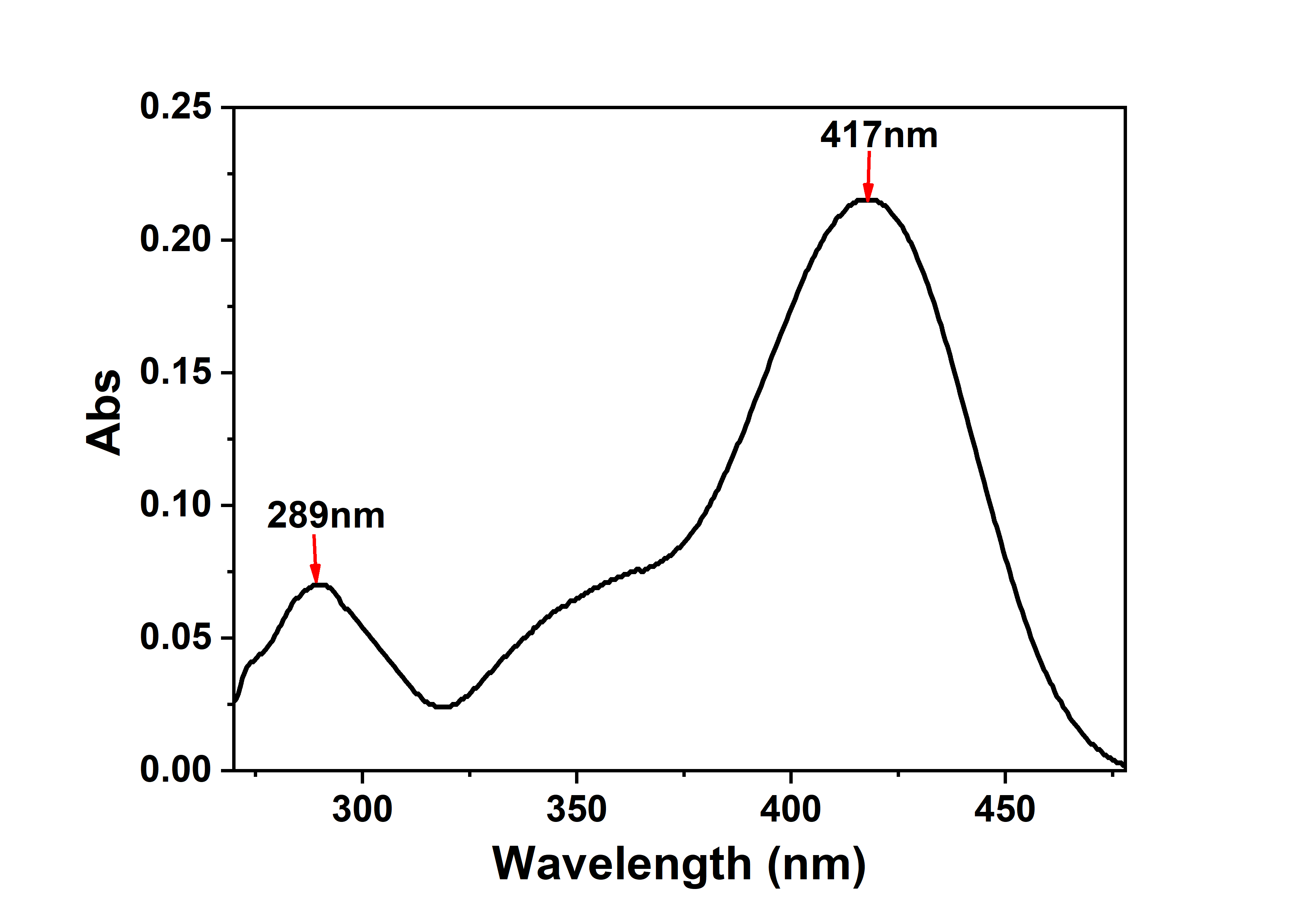


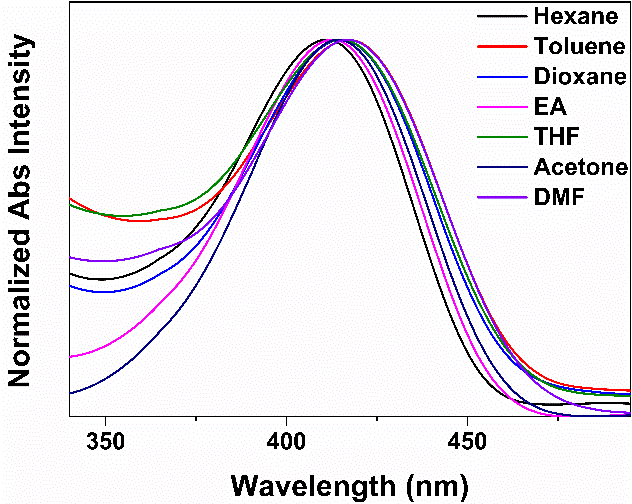


**Fig. S5** Upper: Absorption spectra of **(OMeTPA)_2_**-**Pyr** in DMF; lower: in variant solvents.


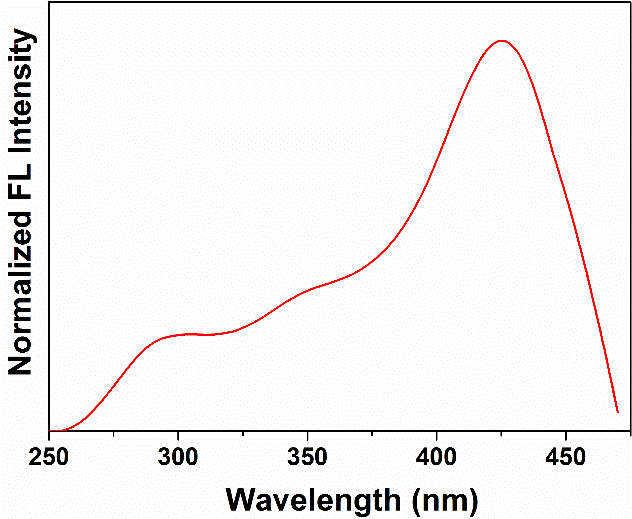


**Fig. S6** Normalized excited fluorescence of **(OMeTPA)_2_**-**Pyr** (2×10^−6^M) in DMF.


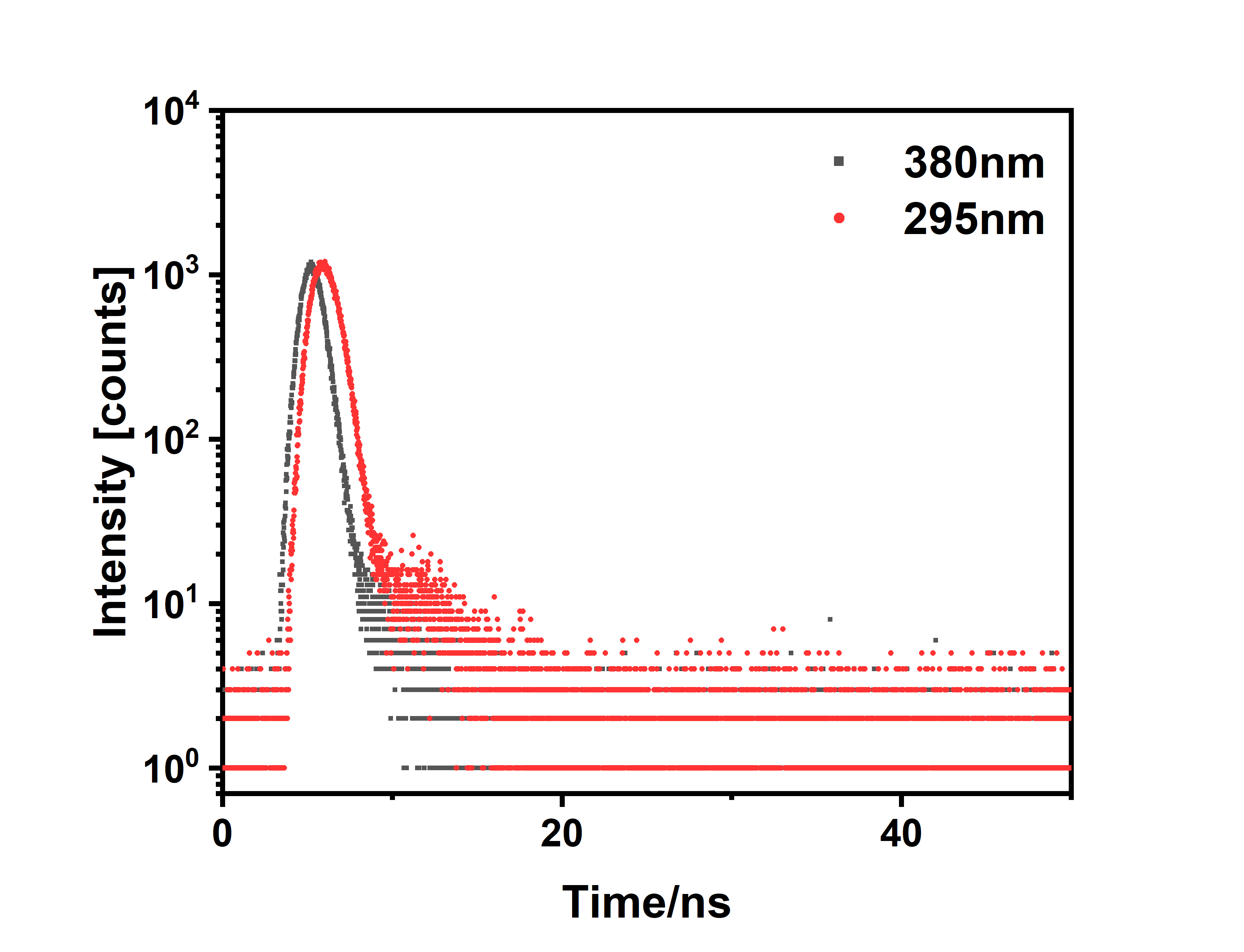


**Fig. S7** Time-resolved fluorescence decay profile of **(OMeTPA)_2_**-**Pyr** (2×10^−6^M) in DMF with excitation wavelengths at 295nm and 380nm respectively.

| **Solvent** | ***E*_T_(30) *Δf*** | |  | | **λ_abs_**  **(nm)** | **λ_em_**  **(nm)** | **Stokes shift**  **nm/(cm^-1^)** |
| --- | --- | --- | --- | --- | --- | --- | --- |
| Hexane | 31.0 | 0 | | 411 | | 489 | 64/(3080) |
| Toluene | 33.9 | 0.014 | | 417 | | 497 | 72/(3409) |
| 1,4-Dioxane | 36.0 | 0.027 | | 415 | | 499 | 74/(3489) |
| EA | 37.4 | 0.201 | | 413 | | 513 | 88/(4036) |
| THF | 38.6 | 0.210 | | 415 | | 515 | 90/(4112) |
| Acetone | 42.2 | 0.284 | | 414 | | 544 | 119/(5147) |
| DMF | 43.2 | 0.275 | | 417 | | 549 | 124/(5314) |

**Table. S1** Detailed photophysical data of **(OMeTPA)_2_**-**Pyr** in variant solvents. Abbreviations: λ_abs_=absorption maximum, λ_em_=emission maximum, *Δf* = orientation polarizability, *E*_T_(30) = solvent polarity parameters.


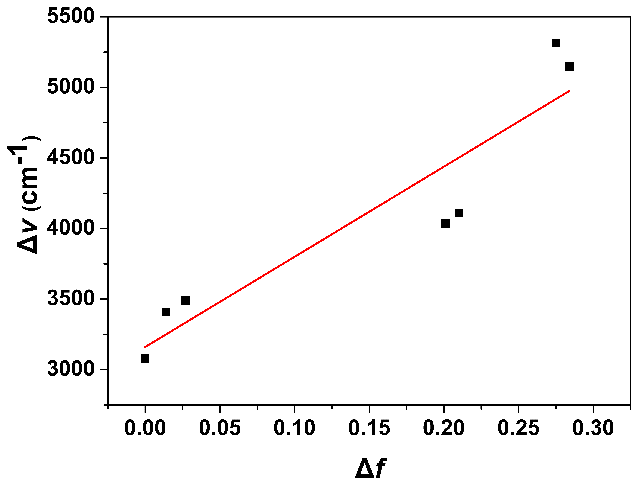


**Fig. S8** Lippert-Mataga plot of Stokes shift (*Δν*) versus the orientation polarization (*Δf*) of solvent media for **(OMeTPA)_2_**-**Pyr**.


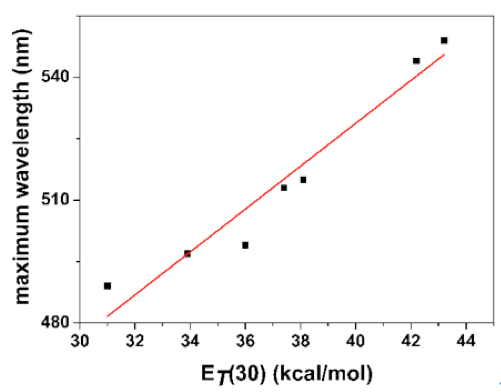


**Fig. S9** Plot of the emission maximum of **(OMeTPA)_2_**-**Pyr** in different solvents versus *E*_T_(30), where *E*_T_(30) was the empirical parameter for solvent polarity.


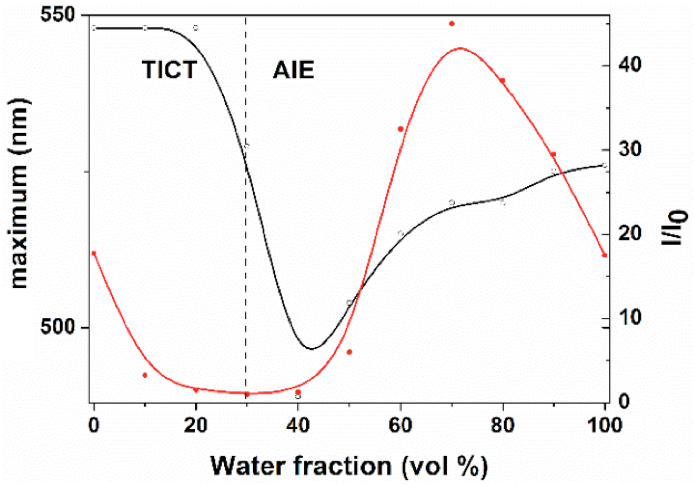


**Fig. S10** Plots of fluorescence maximum and relative intensity (I/I_0_) versus the composition of the DMF/water mixture of **(OMeTPA)_2_**-**Pyr**, where I_0_ was the intensity at 30% *f*_w_. Concentration = 2×10^-6^M, λex = 427 nm.

**Table. S2** Average diameter and PDI of the **(OMeTPA)2**-**Pyr** in DMF/water mixtures with different water fractiona

| ***f*_w_ (%)** | ***d* (nm)** | **PDI** |
| --- | --- | --- |
| 70 | 213 | 0.608 |
| 100 | 19 | 0.305 |

^a^Abbreviations: *d* = average diameter, PDI = polydispersity index


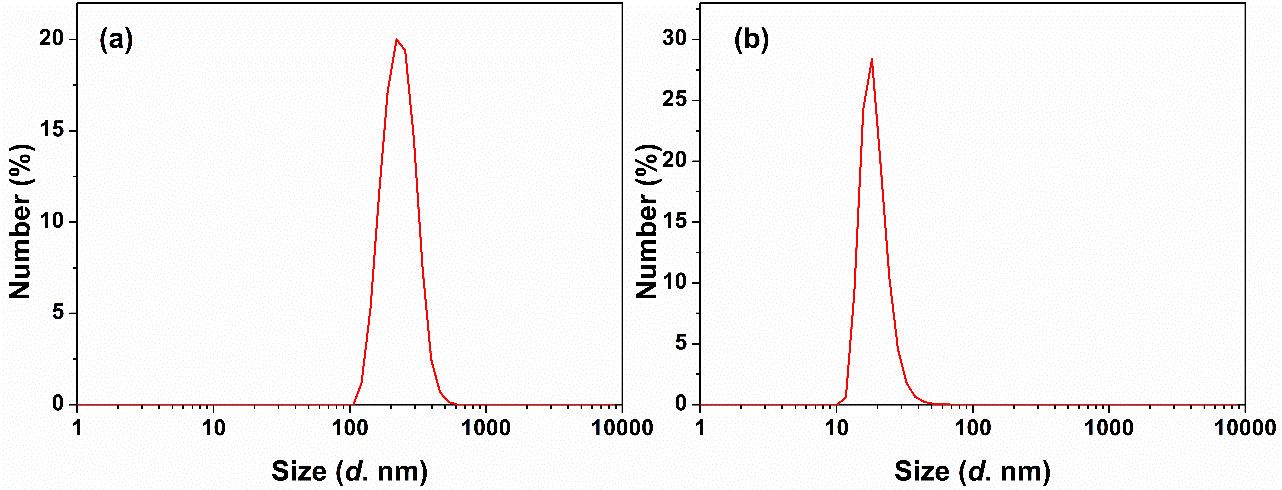


**Fig. S11** Particle size distributions of **(OMeTPA)_2_**-**Pyr** in DMF/water mixtures with (a) *f*_w_ = 70% and (b) *f*_w_ = 100%

**Cell Culture**

4T1 and HeLa cells (Procell, Wuhan, China) were cultured in Dulbecco’s modified Eagle’s media (DMEM) supplemented with 10% fetal bovine serum and100 U mL^−1^ of penicillin–streptomycin at 37 °C in 5% CO_2_.


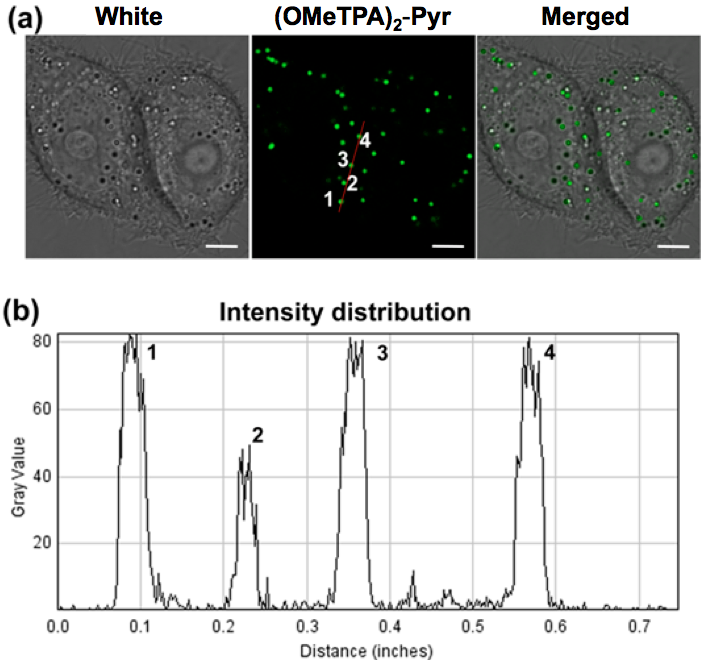


**Fig. S12** Fluorescence images of Hela cells stained with **(OMeTPA)_2_**-**Pyr** (1.5 µg/mL) after 24 hrs incubation and wash. Scale bar, 10 μm. (a) (b) The intensity distribution of **(OMeTPA)_2_**-**Pyr** in Hela cells. Scale bar, 5μm.


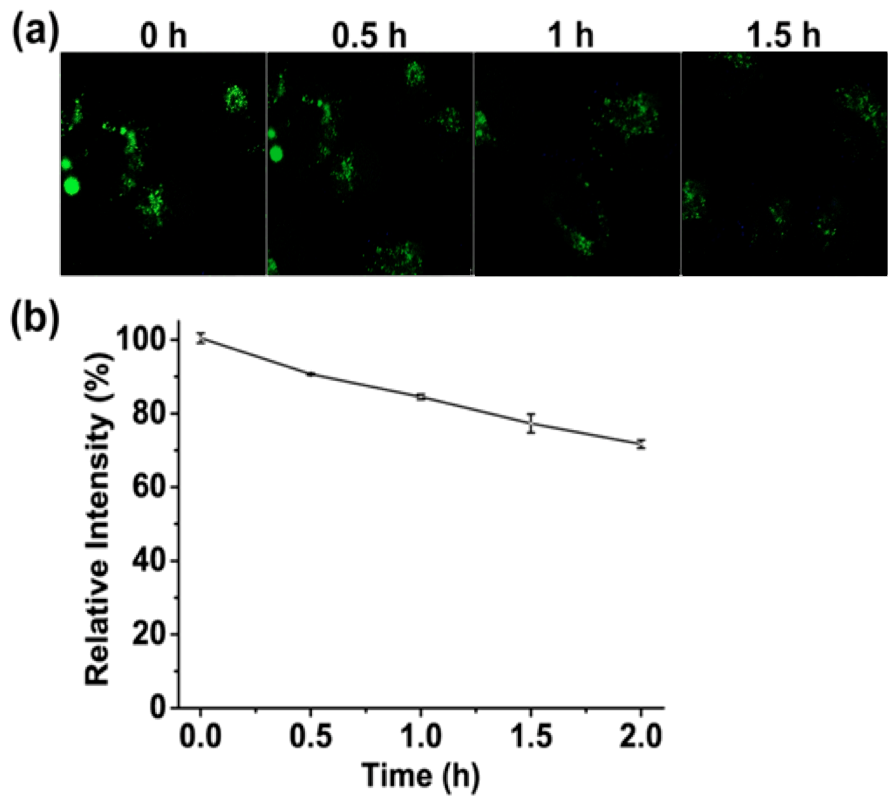


**Fig. S13** (a) Fluorescence imaging of **(OMeTPA)_2_**-**Pyr** (1.5 μg/mL) irradiated by continuous 405 nm laser for 0, 0.5, 1, 1.5 h respectively in live Hela cells. (b) Plots of relative intensity versus irradiation time, where fluorescence intensity was relative to the fluorescence intensity at 0 hours of irradiation.

**
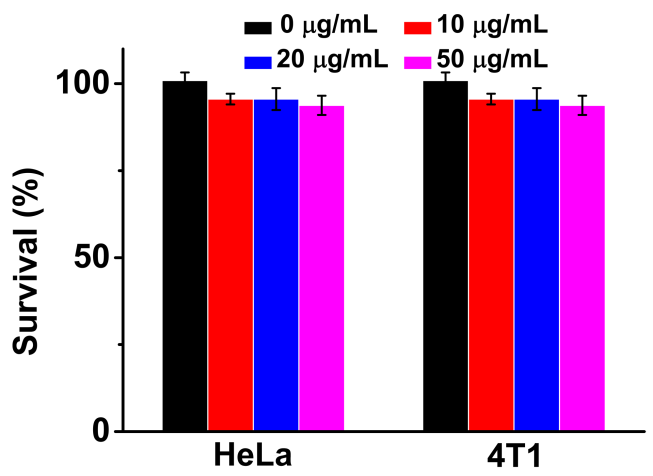
**

**Fig. S14** In vitro cell viability of Hela and 4T1 cells incubated with **(OMeTPA)_2_**-**Pyr** at different concentrations for 24 hrs.
